# Limited Support for Thyroid Hormone or Corticosterone Related Gene Expression as a Proximate Mechanism of Incubation Temperature-Dependent Phenotypes in Birds

**DOI:** 10.3389/fphys.2019.00857

**Published:** 2019-07-05

**Authors:** Sydney F. Hope, Christopher R. Buenaventura, Zahabiya Husain, Sarah E. DuRant, Robert A. Kennamer, William A. Hopkins, Christopher K. Thompson

**Affiliations:** ^1^Department of Fish and Wildlife Conservation, Virginia Tech, Blacksburg, VA, United States; ^2^School of Neuroscience, Virginia Tech, Blacksburg, VA, United States; ^3^Department of Biological Sciences, University of Arkansas, Fayetteville, AR, United States; ^4^Savannah River Ecology Laboratory, University of Georgia, Aiken, SC, United States

**Keywords:** early developmental environment, parental effects, *Aix sponsa*, avian, hippocampus, hypothalamus, glucocorticoids, brain

## Abstract

The conditions that animals experience during early development can have profound consequences for health and fitness. In birds, one of the most important aspects of development is egg incubation temperature. A small decrease in average temperature leads to various impacts on offspring phenotype, such as smaller body sizes, slower growth rates, and less efficient metabolic activity. Little is known, however, about the proximate mechanisms underlying these incubation temperature-induced phenotypic changes. Two important hormones which could play a proximate role are thyroid hormone and corticosterone, which mobilize stored energy reserves and coordinate the normal growth of tissues, particularly in the brain. Previous research shows that circulating blood concentrations of both hormones are influenced by incubation temperature, but the mechanism by which incubation temperature may lead to these changes is unknown. We hypothesized that incubation temperature induces changes in thyroid hormone and corticosterone regulation, leading to changes in expression of hormone-sensitive genes in the brain. To test this, we incubated wood duck (*Aix sponsa*) eggs at three different temperatures within the natural range (35.0, 35.8, and 37.0°C). We measured mRNA expression of thyroid hormone-related neuroendocrine endpoints (deiodinase 2/3, thyroid hormone receptor α/β, neural regeneration related protein, and Krueppel-like factor 9) in newly hatched ducklings and corticosterone-related neuroendocrine endpoints (mineralocorticoid receptor, glucocorticoid receptor, cholecystokinin, and brain-derived neurotrophic factor) in 15 day-old ducklings using qPCR on brain tissue from the hippocampus and hypothalamus. Contrary to our predictions, we found that mRNA expression of thyroid hormone-related endpoints in both brain areas were largely unaffected by incubation temperature, although there was a trend for an inverse relationship between mRNA expression and incubation temperature for several genes in the hypothalamus. We also found that mineralocorticoid receptor mRNA expression in the hypothalamus was lower in ducklings incubated at the low relative to the high temperatures. This study is the first to evaluate the effects of incubation temperature on mRNA expression of neuroendocrine endpoints in the developing avian brain and suggests that these particular endpoints may be largely resistant to changes in incubation temperature. Thus, further research into the proximate mechanisms for incubation temperature-induced developmental plasticity is needed.

## Introduction

During early development, animals are particularly susceptible to changes in their environment, and these developmental conditions can have long-term consequences for health and fitness (Lindström, 1999; Monaghan, 2008). In some cases, changes in the early developmental environment can induce phenotypic changes that optimize offspring for their current or future environment. This type of developmental programming has been described in both ectothermic and endothermic vertebrates (Salinas and Munch, 2012; Mariette and Buchanan, 2016). Developmental plasticity can lead to negative consequences and disease, however, when there are mismatches between the developmental and adult environment, or when suboptimal developmental conditions lead to suboptimal phenotypes (Monaghan, 2008). On the other hand, some traits, especially those that are essential to survival, are more resistant to changes in the early developmental environment and exhibit little developmental plasticity (Waddington, 1942). Among vertebrates, parental behaviors that influence the early developmental environment play a key role in shaping offspring phenotypes. Currently, the underlying hormonal and neuroendocrinological mechanisms in the developing offspring that are affected by changing developmental conditions and contribute to these phenotypic outcomes are poorly understood.

In birds, one of the most important aspects of the environment during early development is egg incubation temperature. Factors such as weather, clutch size, and parental body condition can influence incubation temperatures both directly and indirectly through impacts on parental incubation behavior (Aldrich and Raveling, 1983; Haftorn and Reinertsen, 1985; Conway and Martin, 2000; Coe et al., 2015; Hope et al., 2018a). Thus, egg temperatures can vary substantially both among and within avian nests (Reid et al., 2000; Hepp et al., 2006; Boulton and Cassey, 2012; Coe et al., 2015; Hope et al., 2018a). Importantly, subtle differences (<1°C) in average incubation temperature result in a wide array of phenotypic changes in avian offspring (DuRant et al., 2010, 2011, 2012a,b, 2013a, 2014; Nord and Nilsson, 2011, 2016; Hepp and Kennamer, 2012; Hepp et al., 2015; Berntsen and Bech, 2016). In some cases, this developmental plasticity may be beneficial for offspring. For example, low incubation temperatures produce individuals that have slower growth rates (DuRant et al., 2010; Nord and Nilsson, 2011) and more proactive behaviors (Hope et al., 2018b), which may be advantageous in environments with low resource availability. However, in many cases, low incubation temperatures result in phenotypes that are likely not adaptive, such as reduced thermoregulatory ability (DuRant et al., 2012b, 2013a), reduced ability to fledge from the nest (Hope et al., 2019) and, notably, reduced survival (Hepp and Kennamer, 2012; Berntsen and Bech, 2016; Nord and Nilsson, 2016).

Although the effects of incubation temperature on avian phenotype are well-documented (DuRant et al., 2013b), little is known about the proximate mechanisms underlying these incubation temperature-induced phenotypic changes. Two important hormones that could play a proximate role are thyroid hormones and glucocorticoids. Both thyroid hormones (McNabb and King, 1993; Morreale de Escobar et al., 2004; McNabb, 2006) and glucocorticoids (McEwen, 2001; Kanagawa et al., 2006) are essential for neurogenesis and coordinating the normal growth of tissues in the developing vertebrate brain, which could then have downstream effects on other aspects of offspring phenotype. Indeed, altered levels of both thyroid hormones (Pop et al., 1999; Boelaert and Franklyn, 2005; Ahmed et al., 2008) and glucocorticoids (Maniam et al., 2014) during early development are related to disease and physiological and behavioral disorders in vertebrates. Importantly, incubation temperature is related to circulating levels of both thyroid hormone (DuRant et al., 2014) and corticosterone (DuRant et al., 2010), the major avian glucocorticoid, in juvenile birds. The mechanism by which incubation temperature leads to these changes in hormone levels, or how these changes in hormone levels may have lasting impacts on phenotype, however, is unknown. One possible underlying mechanism may be that incubation temperature influences the development of the major hormonal axes associated with these two hormones (i.e., hypothalamic-pituitary-thyroid [HPT] axis and hypothalamic-pituitary-adrenal [HPA] axis). For example, incubation temperature may induce changes in the expression of genes involved in the regulation of these two hormones, particularly in developing brain tissue. Furthermore, incubation temperature-induced changes in circulating hormones are likely to induce changes in the expression of hormone-sensitive genes in the developing brain, which will then have an impact on physiology and behavior, even if changes in hormone levels are transient. A better understanding of these mechanisms is critical for assessing the impact of altered environmental conditions on developing organisms.

In this study, we hypothesized that incubation temperature induces changes in thyroid hormone and corticosterone regulation, leading to changes in the expression of thyroid hormone and corticosterone sensitive genes that are important for normal brain development. Some of the genes examined are also involved in regulating these hormone systems, particularly in the hypothalamus. To test this hypothesis, we incubated wood duck (*Aix sponsa*) eggs at three different temperatures within the natural range (35.0, 35.8, and 37.0°C; DuRant et al., 2013b). To measure gene expression, we used quantitative PCR (qPCR) on duckling brain tissue from the hippocampus and hypothalamus. We measured mRNA expression of the thyroid hormone-related genes deiodinase 2/3 (DIO2 and DIO3), thyroid hormone receptor α/β (TRα and TRβ), neural regeneration related protein (NREP), and Krueppel-like factor 9 (KLF9) in newly hatched ducklings. We measured mRNA expression of the corticosterone-related genes mineralocorticoid receptor (MR), glucocorticoid receptor (GR), cholecystokinin (CCK), and brain-derived neurotrophic factor (BDNF) in 15 day-old ducklings. Because previous research shows that ducklings incubated at lower temperatures have lower levels of the more active form of thyroid hormone (T_3_; DuRant et al., 2014) and higher baseline and stress-induced corticosterone levels (DuRant et al., 2010) than those incubated at higher temperatures, we expected that mRNA expression would be altered by incubation temperature. Specifically, we predicted that the expression of genes that are stimulated by thyroid hormone (TRβ, DIO3, NREP, and KLF9) would be the lowest (Hu et al., 2016; Thompson and Cline, 2016), and genes that are downregulated by thyroid hormone signaling (TRα and DIO2) would be the highest in ducklings incubated at the lowest temperature (Bernal, 2007; Duarte-Guterman and Trudeau, 2010). Further, we predicted that the expression of genes related to the regulation and negative feedback of corticosterone (MR and GR) and those that are normally suppressed by corticosterone (BDNF) would be the lowest, and that genes that are related to HPA axis stimulation (CCK) would be highest in ducklings incubated at the lowest temperature (Murakami et al., 2005; Liu et al., 2010; Datson et al., 2012).

## Materials and Methods

### Study Species and Site

The wood duck is a common North American duck that nests in tree cavities and nest boxes (Hepp and Bellrose, 2013). The wood duck breeding season begins in mid-February and lasts until mid-July, and re-nesting is common (Hepp and Bellrose, 2013). The female incubates the eggs without help from the male, and eggs hatch after ∼30 days of incubation (Hepp and Bellrose, 2013). Females spend most of the day incubating but usually take two 1–2 h recesses each day to forage (Manlove and Hepp, 2000). Incubation temperature varies among and within populations (Bellrose and Holm, 1994; Manlove and Hepp, 2000; Hepp et al., 2006), and within nests (Hope et al., 2018a). Incubation temperature is lower and more variable as clutch size increases (Hope et al., 2018a). Clutch sizes (average = 12 eggs; Bellrose and Holm, 1994) are variable within populations and can reach >40 eggs (Morse and Wight, 1969) due to conspecific brood parasitism (Semel and Sherman, 1986; Semel et al., 1988; Roy Nielsen et al., 2006). Offspring are precocial and can feed themselves once they leave the nest (Bellrose and Holm, 1994).

### Egg Collection and Incubation

Our methods for egg collection and incubation are described fully in Hope et al. (2018b). Briefly, we collected unincubated wood duck eggs from nest boxes at the Department of Energy’s Savannah River Site in South Carolina (33.1°N, 81.3°W) in March 2015. We incubated eggs in Grumbach incubators (model BSS 420, Asslar, Germany) at Virginia Tech. Eggs were incubated at three different average temperatures (35.0, 35.8, and 37.0°C). We chose these temperatures because they are within the natural range for wood ducks and have been shown to produce variation in a wide array of duckling phenotypes (DuRant et al., 2013b and references therein), including corticosterone levels (DuRant et al., 2010), thyroid hormone levels (DuRant et al., 2014), and behavior (Hope et al., 2018b). Incubators had two daily 75 min cool-down periods at 8:15 and 18:30 h to mimic natural conditions (Manlove and Hepp, 2000). During these cooling periods, the incubators turned off for 75 min, which produced different minimum temperatures for each treatment (average minimum temperatures ± SD for each treatment: 35.0°C: 32.9 ± 0.85°C; 35.8°C: 33.8 ± 0.89°C; 37.0°C: 33.5 ± 0.95°C). Temperature data from *i*Buttons^®^ (Hygrochron, Maxim Integrated, CA, United States) within the incubators confirmed that they maintained the above mentioned average temperatures.

### General Husbandry

For this study, 30 ducklings from 18 nests (8 incubated at 35.0°C, 11 at 35.8°C, and 11 at 37.0°C) were sacrificed at day 0, and 35 ducklings from 27 nests (11 incubated at 35.0°C, 12 at 35.8°C, and 12 at 37.0°C) were sacrificed on day 15 to collect brain tissue. Once hatched, day 0 ducklings were placed in a covered cage with a 50W infrared heat lamp with other similar-aged ducklings for <10 h, until they were euthanized. Day 15 ducklings were housed in cages with *ad lib* food and water, and underwent a series of behavioral trials as part of a different experiment. Complete animal husbandry methods for these ducklings are described in Hope et al. (2018b).

### Tissue Collection

Ducklings were humanely euthanized via carbon dioxide asphyxiation and cervical dislocation. Immediately following euthanasia, whole brains were extracted from ducklings and flash-frozen on dry ice (≤12 min from start of euthanasia until brain completely frozen). Brains were then stored at -80°C until sectioning. Using a cryostat, brains were sectioned until the hippocampus and hypothalamus regions were visible. We took two punches from each brain region of each bird, and these two punches were pooled for each bird, producing one sample per brain region per bird. Punches were taken from only one side (i.e., right or left) of each brain, and we alternated which brain region we sampled for each bird to test for hemispheric differences. Punches were immediately placed in Trizol and frozen on dry ice. Samples were stored at -80°C until analysis.

### Quantifying mRNA Expression

We investigated thyroid hormone-related mRNA expression in day 0 ducklings because thyroid hormone is important during early development and, in a previous study, wood duck ducklings incubated at different temperatures displayed different levels of circulating thyroid hormone on day 0, but not on day 4 or 10 (DuRant et al., 2014). We investigated corticosterone-related gene expression in day 15 ducklings because wood duck ducklings incubated at different temperatures display differences in corticosterone levels at day 9 (DuRant et al., 2010), and because post-mortem tissue samples were available at day 15 after a previous study (Hope et al., 2018b). Further, some animals undergo a refractory period shortly after hatch, where HPA axis function is dampened and individuals are unresponsive to stressors (Sapolsky and Meaney, 1986), thus we did not expect to find meaningful results for the HPA axis in day 0 ducklings. Although effects of incubation temperature on circulating levels of corticosterone appear to be absent by day 15 (Hope et al., 2018b), there are still lasting effects on behavior until day 15 (Hope et al., 2018b), and thus we postulated that transient elevations of corticosterone until day 9 would lead to changes in the HPA axis, including altered gene expression, that would be apparent at day 15.

We extracted RNA per the manufacturer’s instructions for Trizol, measured the amount of RNA extracted on a NanoDrop, and reverse transcribed the mRNA using the iScript kit (Bio-Rad, CA, United States), using 1 ug of RNA per reaction. Tissue samples from each duckling were run individually. We performed qPCR using 8 ng cDNA per reaction using the iTaq Universal SYBR Green Supermix kit (Bio-Rad) on a Bio-Rad CFX384 thermocycler. All reactions were done with technical triplicates; outlier reactions (deviations more than 1.5 times the SD from the mean) within a set of triplicates were removed from analysis. We used a two-step reaction with a 10 s 95° melt step, followed by a 30 s 60° annealing and an extension step for 40 cycles, with fluorescence measured at the end of every 60° step. At the end of 40 cycles, we evaluated the melt curves for secondary products; none of the primers used in this study generated secondary products. We designed primers by blasting the genes of interest from mallard (*Anas platyrhynchos*) genome to the domestic goose (*Anser cygnoides domesticus*) genome and selecting regions of high homology using NCBI primer blast online tool. A list of primers is found in Table 1. Primer pairs were selected using PCR against wood duck cDNA and running out the product on a gel to ensure each primer pair generated the expected product size, only one product, and no primer-dimer production.

**Table 1:** Table of primers used for quantitative PCR.

Category	Gene	Gene name	Primer direction	Primer sequence	Amplicon size (bp)
**Reference**	ACTB	Actin beta	For	GCAGATGTGGATCAGCAAGC	98
			Rev	AGGGTGTGGGTGTTGGTAAC	
	GAPDH	Glyceraldehyde-3-phosphate dehydrogenase	For	TCTGGCAAAGTCCAAGTGGT	99


			Rev	CCGGAAGTGGCCATGAGTAG	
**Thyroid Hormone**	DIO2	Deiodinase 2	For	GATGCGTCAACAGGTGTGTC	118


			Rev	GTGTTCTCCTGCAATGATCTGA	
	DIO3	Deiodinase 3	For	GCGAGCTTTCGAGCAAGATG	82
			Rev	ACAGTATCGACAGAGCGTGG	
	KLF9	Kruppel-like factor 9	For	TGGTTCTTCAGAGCACTGCG	123
			Rev	CCCGCTTTTTCTCATCCAGC	
	NREP	Neuronal regeneration related protein	For	TGTGATGCTGCCACAGGATT	82
			Rev	CCATCAGAGCTACCTTGCCA	
	TRα	Thyroid hormone receptor α	For	TCTTCGACCTGGGCAAATCC	90
			Rev	TACCTGAGGACATGAGCAGC	
	TRβ	Thyroid hormone receptor β	For	CCACAGAACTCTTCCCTCCG	121
			Rev	TGAGAAGAGCTGGGCAATGG	
**Corticosterone**	GR	Nuclear receptor subfamily 3 group C member 1	For	GACCTCTCCAAGGCAGTGTC	138


			Rev	CAGGAGCCTGAAGTCCGTTT	
	MR	Nuclear receptor subfamily 3 group C member 2	For	ATTCGGAGGAAGAACTGCCC	79


			Rev	TGGACTTTCGGGCTCCTAGA	
	CCK	Cholecystokinin	For	GGGGCTCACACAATGACAAC	101
			Rev	TTTCAGGGGGCCAGTAGACA	
	BDNF	Brain derived neurotrophic factor	For	ATGTTCCACCAAGTGAGAAGAG	133
			Rev	GACCTGGGTAAGCCAAGCTG	


To evaluate the suitability of GAPDH and ACTB as reference genes, we used two basic forms of analysis. First, we examined if raw Ct (the cycle at which florescence intensity reached threshold) for each reference gene significantly varied across temperatures in each brain area. We found that temperature did not affect ACTB and GAPDH expression (*p* = 0.55 and 0.89, respectively). We also assessed reference gene stability using the geNorm and NormFinder analysis tools in Excel (Microsoft). GAPDH and ACTB met the criteria to be used as stable reference genes in these experimental conditions. We then used the ΔΔCt method to calculate change in expression relative to the average of the reference genes (GAPDH and ACTB). Last, we normalized expression data using the results from the 35°C hypothalamus group as a baseline. Fold change is a 10-base fold change because results were transformed using a square exponent (i.e., 2^-ΔΔ*Ct*^).

### Statistical Analyses

We used GraphPad Prism to analyze our data for statistical differences. Data sets from individual brain areas were evaluated with one way ANOVA with temperature as the independent factor for each brain region and at each time point, and *post hoc* comparisons were made with Tukey’s multiple comparisons test. We also tested for hemispheric (i.e., left vs. right) differences in mRNA expression in each brain region using one way ANOVAs. We were unable to use repeated measures ANOVA to include brain region as an additional factor because the data set was not completely balanced (i.e., some punches from some brain areas in some animals had poor RNA extraction/quality and therefore could not be compared using within-animals comparisons). However, we were able to make general observations about overall expression patterns across brain regions without respect to incubation temperature. Sample sizes for each analysis (reflecting individuals that were excluded due to poor RNA extraction/quality) are reported in Table 2.

**Table 2:** Summary of statistics.

Category	Gene	Gene name	Hippocampus			Hypothalamus		
			***F***	***N***	***p*-value**	***F***	***N***	***p*-value**
**Thyroid Hormone**	DIO2	Deiodinase 2	0.2634	29	0.7705	0.9964	28	0.3834
	DIO3	Deiodinase 3	0.4098	29	0.668	0.4087	28	0.6689
	KLF9	Kruppel-like factor 9	0.7097	29	0.5011	1.643	28	0.2135
	NREP	Neuronal regeneration related protein	0.1175	29	0.8897	1.689	28	0.2051
	TRα	Thyroid hormone receptor α	0.2331	29	0.7937	1.183	28	0.323
	TRβ	Thyroid hormone receptor β	0.2719	29	0.7641	0.0418	28	0.9591
**Corticosterone**	GR	Nuclear receptor subfamily 3 group C member 1	0.08396	28	0.9197	0.513	33	0.6038
	MR	Nuclear receptor subfamily 3 group C member 2	0.1609	28	0.8523	***5.744***	***32***	***0.0079***
	CCK	Cholecystokinin	0.08543	27	0.9184	1.952	30	0.1609
	BDNF	Brain derived neurotrophic factor	1.727	28	0.1984	1.58	32	0.2232


*One way ANOVA across 3 temperature treatments within each brain region. Tukey’s multiple comparisons test as a *post hoc* test for ANOVAs with *p* < 0.05.*

## Results

### mRNA Expression of Thyroid Hormone-Associated Neuroendocrine Endpoints

**FIGURE 1:**
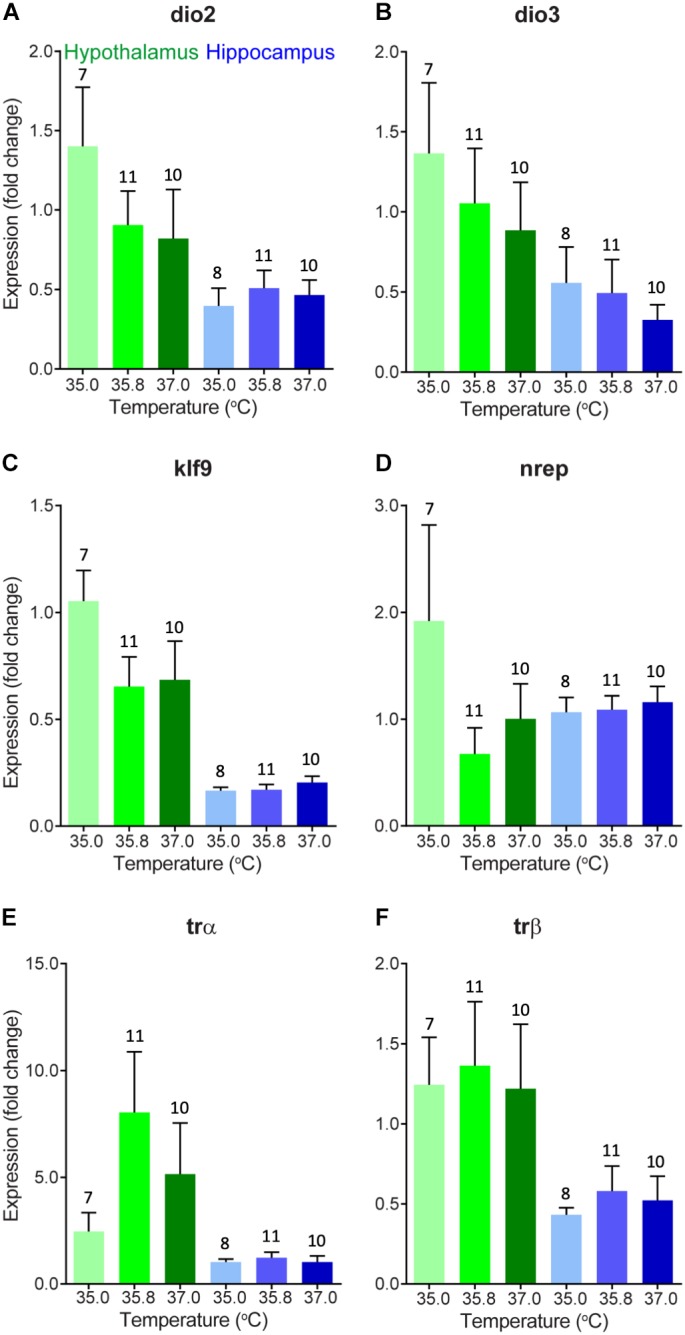
Gene expression (mean fold change compared to 35°C hypothalamus group as a baseline) of **(A)** deiodinase 2, **(B)** deiodinase 3, **(C)** Krueppel-like factor 9, **(D)** neural regeneration related protein, **(E)** thyroid hormone receptor α, and **(F)** thyroid hormone receptor β in the hypothalamus (green) and hippocampus (blue) of newly hatched (day 0) wood duck ducklings incubated at three different incubation temperatures. Gene expression was measured using quantitative PCR of brain tissue. Fold change is a 10-base fold change because results were transformed using a square exponent (i.e., 2^-ΔΔ*Ct*^). Error bars indicate standard error. Numbers above bars indicate sample size.

We evaluated changes in gene expression in six different thyroid hormone-related genes of interest in 0 day-old ducklings that were incubated at three different temperatures (Figure 1). A summary of these statistical results can be found in Table 2. In short, we did not observe statistically significant differences in gene expression across incubation temperatures in the two brain areas examined. There was a trend of elevated expression at lower incubation temperatures in the hypothalamus of several thyroid hormone-related neuroendocrine endpoints (Figure 1), but none of these results were statistically different.

### mRNA Expression of Corticosterone-Associated Neuroendocrine Endpoints

**FIGURE 2:**
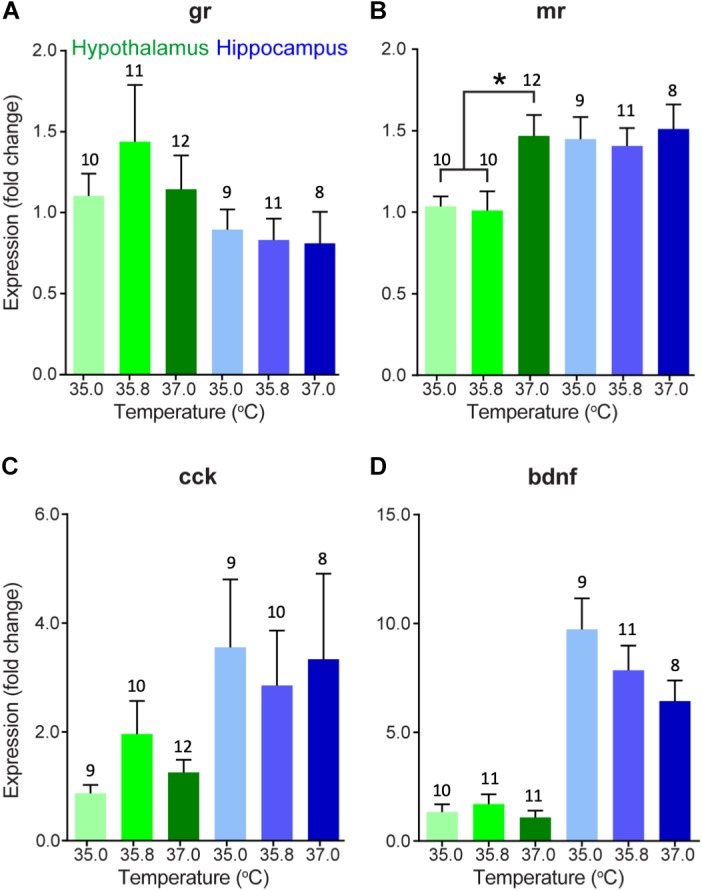
Gene expression (mean fold change compared to 35°C hypothalamus group as a baseline) of **(A)** glucocorticoid receptor, **(B)** mineralocorticoid receptor, **(C)** cholecystokinin, and **(D)** brain-derived neurotrophic factor in the hypothalamus (green) and hippocampus (blue) of 15 day-old wood duck ducklings incubated at three different incubation temperatures. Gene expression was measured using quantitative PCR of brain tissue. Fold change is a 10-base fold change because results were transformed using a square exponent (i.e., 2^-ΔΔ*Ct*^). Error bars indicate standard error. Asterisk indicates significant effect of incubation temperature (*p* < 0.05), as determined by a one-way ANOVA and Tukey HSD test. Numbers above bars indicate sample size.

We also evaluated changes in gene expression in four different corticosterone-related genes of interest in two different brain areas in 15 day-old ducklings that were incubated at three different temperatures (Figure 2). A summary of statistical results can be found in Table 2. We found that expression of MR was significantly lower in the hypothalamus at 35.0 and 35.8°C relative to 37.0°C (*p* < 0.01), with expression at about 2/3 of the levels seen at 37.0°C. Temperature did not appear to affect gene expression in the three other genes evaluated (GR, CCK, and BDNF).

### Comparisons Among Brain Regions

There were several general expression patterns observed in our study across brain regions without respect to temperature. First, we found that, in general, expression of thyroid hormone-related genes was substantially higher in the hypothalamus than in the hippocampus, particularly KLF9 (Figure 1C) and the receptors of thyroid hormone, TRα, and TRβ (Figure 1E,F) in 0 day-old ducklings. We also found that BDNF expression was much higher in the hippocampus than in the hypothalamus in 15 day-old ducklings (Figure 2D). We found that MR mRNA expression was significantly higher in the right hemisphere compared to the left hemisphere (*p* = 0.015) of the hypothalamus, but there were no other hemispheric differences in other genes/brain regions (all *p* ≥ 0.29).

## Discussion

In this study, we investigated whether incubation temperature influences the expression of genes related to the regulation of thyroid hormone and corticosterone in the developing avian brain. We found that a 1–2°C decrease in average incubation temperature resulted in lower mRNA expression of MR in the hypothalamus of 15 day-old wood duck ducklings, which may have implications for regulation of corticosterone at baseline levels. Aside from this, we found no other significant differences in mRNA expression among incubation temperature treatments. This is surprising, considering the wide array of differences in phenotypes that are manifested as a result of small changes in incubation temperature in wood ducks and other species (DuRant et al., 2010, 2011, 2012a,b, 2013a, 2014; Nord and Nilsson, 2011, 2016; Hepp and Kennamer, 2012; Hepp et al., 2015; Berntsen and Bech, 2016), and suggests that altered expression of these particular genes at this developmental stage is likely not the mechanism underlying incubation temperature-induced developmental plasticity. On the contrary, this suggests that the expression of these genes in the brain may be robust to changes in the developmental environment, including fluctuations in circulating levels of thyroid hormone and corticosterone.

We found that MR mRNA expression in the hypothalamus was lower in ducklings incubated at 35.0 and 35.8°C than those incubated at 37.0°C. MR has a higher affinity for corticosterone than GR and thus, mediates most of the effects of corticosterone at baseline levels (Kloet et al., 1998). Therefore, differences in MR mRNA expression may indicate the ability of ducklings to regulate normal rhythms of corticosterone levels (e.g., diurnal and circadian) and may underlie differences in baseline corticosterone levels that are seen in ducklings incubated at different temperatures (DuRant et al., 2010). Indeed, our result is consistent with what we would expect given the results from a previous study where ducklings incubated at low temperatures had higher corticosterone levels than those incubated at higher temperatures (DuRant et al., 2010). When circulating levels of corticosterone are high, we would expect that, in order to maintain homeostasis, the brain would respond by decreasing the expression of receptors to decrease its sensitivity to corticosterone. Because the effect of incubation temperature on circulating corticosterone levels is apparent on day 9 (DuRant et al., 2010), but appears to be absent by day 15 (Hope et al., 2018b), the change in MR mRNA expression that we find in this study at day 15 may be a long-term consequence of a transient surge in corticosterone prior to day 15. Given that we found this result in the hypothalamus but not in the hippocampus suggests that the paraventricular nucleus (located in the hypothalamus) has increased MR mRNA expression and may have begun to modulate circulating levels of corticosterone in the animals incubated at the highest temperature but not those incubated at the lower two temperatures. Interestingly, we did not find a significant effect of incubation temperature on GR mRNA expression in either brain region, which is important for regulation of corticosterone at stress-induced levels and negative feedback (Kloet et al., 1998). Thus, it is possible that decreased MR mRNA expression in just the hypothalamus does not translate into major differences in phenotype in these ducklings. Further research is needed to determine any causal relationships among gene expression and aspects of health and phenotype. For instance, experiments using *in situ* hybridization would shed light on any anatomical changes in expression that might be masked when using qPCR. In addition, experiments to assess rates of neurogenesis in the hippocampus may reveal an effect of incubation temperature-induced changes in corticosterone levels.

Although mRNA expression of most genes analyzed did not appear to be affected by differences in incubation temperature, we did identify some apparent differences in the expression of some genes between the hippocampus and the hypothalamus. Our results show that BDNF mRNA expression is very high in the hippocampus of day 15 ducklings relative to the expression in the hypothalamus. The hippocampus is likely undergoing substantial development at this stage, and elevated BDNF mRNA expression would contribute to cellular changes necessary for a functional hippocampus. Levels of neurogenesis are generally very high in the developing hippocampus of young Japanese quail chicks relative to older animals (Nkomozepi et al., 2018), and the same is to be expected in the wood duck hippocampus. Furthermore, there is relatively little neurogenesis in the young quail hypothalamus (Nkomozepi et al., 2018), which is consistent with our observations of higher BDNF expression in the hippocampus relative to hypothalamus.

In general, HPT-axis associated mRNA expression was higher in the hypothalamus than in the hippocampus in day 0 ducklings. This suggests that relative sensitivity to and ongoing molecular regulation of thyroid hormone signaling is likely higher in the hypothalamus than in the hippocampus at this stage in development. The elevated expression of HPT-axis associated neuroendocrine endpoints may suggest that the relative contribution of thyroid hormone to cellular and molecular mechanisms of brain development is higher in the hypothalamus and/or in the paraventricular nucleus, which regulates thyroid hormone synthesis by expression of thyrotropin releasing hormone already at this stage in development. Given the small size of regions of the hypothalamus, punches of heterogeneous populations of neurons across animals may have contributed to the variability observed in our results and masked specific differences observed in the trends noted above. Future experiments can address this issue using either single cell transcriptomics or laser capture of specific hypothalamic brain regions. In addition, circulating cerebral spinal fluid (CSF) levels of transthyretin (TTR), a choroid plexus-derived thyroid hormone distributor protein (Dickson et al., 1986; Richardson et al., 2015), may also be affected by temperature, which could allow for compensation of different circulating levels of thyroid hormone in plasma (DuRant et al., 2014). Future experiments could address this by directly measuring levels of TTR in CSF or examining the expression profile of TTR in the choroid plexus. Last, monocarboxylate transporter 8 (MCT8) transports thyroid hormone across the cell membrane, including transfer of thyroid hormone across the choroid plexus (Roberts et al., 2008). Thus, changes in expression of MCT8 or another MCT such as MCT10 in the hypothalamus, hippocampus, or choroid plexus, could be another compensatory mechanism for temperature-derived changes in circulating levels of thyroid hormone.

The aim of this study was to investigate if differences in HPT or HPA axis function, specifically the mRNA expression of genes related to thyroid hormone or corticosterone regulation, may be a mechanism underlying the pervasive effects of incubation temperature on avian offspring phenotype, health, and fitness (DuRant et al., 2013b). We found some evidence that incubation temperature influenced corticosterone-related gene expression (i.e., MR) but little evidence that it affected thyroid hormone-related gene expression. Thus, the mechanism by which developmental egg temperature leads to changes in phenotype is still largely unknown.

## Data Availability

Datasets are available on request. The raw data supporting the conclusions of this manuscript will be made available by the authors, without undue reservation, to any qualified researcher.

## Ethics Statement

This study was carried out in accordance with the recommendations of the Virginia Tech Institutional Animal Care and Use Committee (VT IACUC). The protocol was approved by the VT IACUC.

## Author Contributions

SH, SD, RK, WH, and CT conceived the idea of the study. RK provided access to the field site and study system. SH conducted the field and captive animal work. SH, CB, ZH, and CT conducted the laboratory work. CT conducted the statistical analyses. SH and CT wrote the manuscript. All authors approved the final version of the manuscript.

## Conflict of Interest Statement

The authors declare that the research was conducted in the absence of any commercial or financial relationships that could be construed as a potential conflict of interest.
